# Molecular detection of rifampin and isoniazid resistance to guide chronic TB patient management in Burkina Faso

**DOI:** 10.1186/1471-2334-9-142

**Published:** 2009-08-28

**Authors:** Paolo Miotto, Nuccia Saleri, Mathurin Dembelé, Martial Ouedraogo, Gisèle Badoum, Gabriele Pinsi, Giovanni B Migliori, Alberto Matteelli, Daniela M Cirillo

**Affiliations:** 1 Emerging Bacterial Pathogens Unit, San Raffaele Scientific Institute, Milan, Italy; 2 Institute of Infectious and Tropical Diseases, Brescia University, Brescia, Italy; 3 National Tuberculosis Program, Ministry of Health, Ouagadougou, Burkina Faso; 4 Department of Pulmonary Care, "Yalgado" National Hospital, University of Ouagadougou, Ouagadougou, Burkina Faso; 5 WHO Collaborating Centre for Tuberculosis and Lung Disease, S. Maugeri Foundation Care and Research Institute, Tradate, Italy

## Abstract

**Background:**

Drug-resistant tuberculosis (DR-TB) is considered a real threat to the achievement of TB control. Testing of mycobacterial culture and testing of drug susceptibility (DST) capacity are limited in resource-poor countries, therefore inadequate treatment may occur, favouring resistance development. We evaluated the molecular assay GenoType^® ^MTBDR*plus *(Hain Lifescience, Germany) in order to detect DR-TB directly in clinical specimens as a means of providing a more accurate management of chronic TB patients in Burkina Faso, a country with a high TB-HIV co-infection prevalence.

**Methods:**

Samples were collected in Burkina Faso where culture and DST are not currently available, and where chronic cases are therefore classified and treated based on clinical evaluation and sputum-smear microscopy results. One hundred and eight chronic TB patients (sputum smear-positive, after completing a re-treatment regimen for pulmonary TB under directly observed therapy) were enrolled in the study from December 2006 to October 2008. Two early morning sputum samples were collected from each patient, immediately frozen, and shipped to Italy in dry ice. Samples were decontaminated, processed for smear microscopy and DNA extraction. Culture was attempted on MGIT960 (Becton Dickinson, Cockeysville, USA) and decontaminated specimens were analyzed for the presence of mutations conferring resistance to rifampin and isoniazid by the molecular assay GenoType^® ^MTBDR*plus*.

**Results:**

We obtained a valid molecular test result in 60/61 smear-positive and 47/47 smear-negative patients.

Among 108 chronic TB cases we identified patients who (i) harboured rifampin- and isoniazid-susceptible strains (n 24), (ii) were negative for MTB complex DNA (n 24), and (iii) had non-tuberculous mycobacteria infections (n 13). The most represented mutation conferring rifampin-resistance was the D516V substitution in the hotspot region of the *rpoB *gene (43.8% of cases). Other mutations recognized were the H526D (15.6%), the H526Y (15.6%), and the S531L (9.4%).

All isoniazid-resistant cases (n 36) identified by the molecular assay were carrying a S315T substitution in the *katG *gene. In 41.7% of cases, a mutation affecting the promoter region of the *inhA *gene was also detected.

**Conclusion:**

The GenoType^® ^MTBDR*plus *assay performed directly on sputum specimens improves the management of chronic TB cases allowing more appropriate anti-TB regimens.

## Background

Emergence of drug-resistant *Mycobacterium tuberculosis *(MTB) strains is considered a real threat to achieving tuberculosis (TB) control [1-3]. Furthermore, multidrug-resistant TB (MDR-TB) and extensively drug-resistant TB (XDR-TB) require prolonged and expensive chemotherapy, with a decreased cure rate [4-6].

As diagnosis of MDR- and XDR-TB is based on mycobacterial culture and drug susceptibility testing (DST) on liquid or solid media – with results available within weeks to months [7-9] – inadequate treatment as well as further spread of resistant strains and development of super-resistance are likely to occur. Furthermore, standardized and optimised MTB culture and DST procedures require equipped, safe laboratories and trained human resources operating under quality assurance protocols. For all of these reasons, mycobacterial culture and DST capabilities are severely limited in resource-poor countries.

In response to the growing problem of MDR-TB and the threat of an epidemic of XDR-TB, the STOP TB strategy has been revised to include universal access to diagnosis and treatment for all patients with MDR-TB by 2015 [10,11]. This plan calls for accelerated access to rapid testing for rifampin (RIF) resistance in order to improve case detection in all patients with suspected MDR- and XDR-TB. To ensure universal access, new technologies for rapid detection of anti-TB drug resistance in MTB have become a priority, especially in settings with high HIV-prevalence [12-15]. Early detection of MDR-TB and XDR-TB is critical to initiating appropriate treatment, reducing morbidity and mortality, and preventing further transmission of drug-resistant MTB strains.

Although rapid molecular methods are available for detecting drug-resistant TB [13,14,16], the feasibility and cost-effectiveness of large-scale implementation in high-burden low-income settings need to be assessed.

Demonstration projects on the GenoType^® ^MTBDR*plus *assay (Hain Lifesciences GmbH, Nehren, Germany), a polymerase chain reaction (PCR) amplification and reverse hybridization assay which detects RIF and isoniazid (INH) resistance, have been conducted in a high-burden low-income country [17]. This assay detects mutations in the *rpoB *gene for RIF resistance, and in both the *katG *gene and the promoter region of the *inhA *gene for INH resistance, directly from smear-positive sputum within one day [18].

We evaluated the GenoType^® ^MTBDR*plus*, a reverse hybridization line probe assay for which detects DR-TB directly in clinical specimens as a means to provide with the purpose of providing more effective management of chronic TB patients in Burkina Faso, a country with a high TB-HIV co-infection prevalence [19].

## Methods

### Study settings

Samples were collected in Burkina Faso, a country in West Africa with an estimated population of 14.9 million, and an estimated incidence of TB (all forms) of 248/100.000 [19]. In 2008, the prevalence of HIV infection in TB patients in Burkina Faso was 12.4% with a prevalence of HIV among adult population estimated to be 1.6% [20,21].

Culture and DST are not currently available in Burkina Faso and chronic TB cases are classified and treated based on clinical evaluation and sputum smear microscopy results.

The work described in this paper was performed in the framework of the agreement for technical support requested by the National Tuberculosis Programme (NTP) of Burkina Faso (responsible body for TB management in the Country) to the Supranational Reference at the San Raffaele Institute, Milan, Italy and the University of Brescia, Italy. The NTP of Burkina Faso didn't consider ethical approval to be necessary since this action was recommended by the Green Light Committee in order to provide better management to chronic TB patients. All patients were informed and consented to the study with the local physicians.

The NTP has now prioritized the establishment of culture and DST facilities by 2009 and rapid testing by 2010.

### Clinical samples

One hundred and eight patients who met the definition of "chronic pulmonary TB" ("patient who remained sputum smear-positive after completing a re-treatment regimen for pulmonary TB under Directly Observed Therapy (DOT)", [22]) were started on a second line drug regimen using a standardised treatment strategy (Category IV) [23]. The Category IV drug regimen used in Burkina Faso consists of kanamycin, ofloxacin, ethionamide, pyrazinamide and cycloserine for six months, followed by 15 months of ofloxacin, ethionamide and cycloserin.

**Figure 1:**
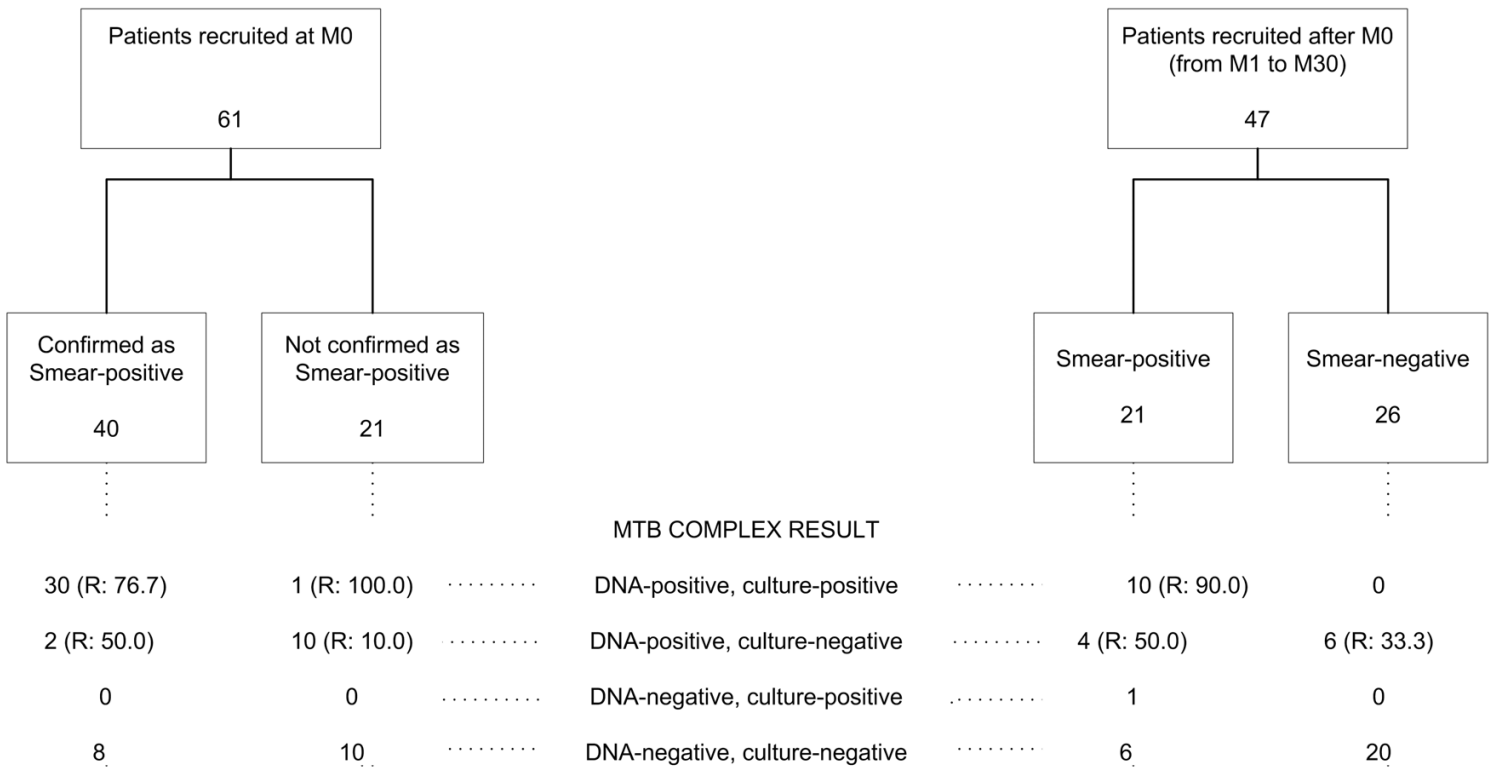
**Culture and GenoType^® ^MTBDR*plus *results in patients enrolled at different times under category IV regimens**. The diagram reports smear microscopy results after the re-testing of samples in Italy and *M. tuberculosis *Complex results by GenoType MTBDR*plus*^® ^and microbiology analyses. Patients are divided in two groups on the basis of the enrolment time in the study (beginning of the therapy, M0; during follow-up, M1–M30). The percentage of resistant cases as "any resistance" is also reported (R).

Patients were enrolled at the two centres for chronic TB patient management in Burkina Faso (Ouagadougou and Bobo-Dioulasso) from December 2006 to October 2008. Sixty-one patients were enrolled at the beginning of a Category IV regimen (Month 0, M0). Forty-seven patients were enrolled at variable times during their Category IV therapy. Twenty-six out the 47 patients enrolled during treatment follow-up were already classified as smear-negative (Figure 1). Two early morning sputum samples were collected from each patient at enrolment into the study, immediately frozen and shipped to Italy in dry ice.

Upon arrival, samples were decontaminated and processed for smear microscopy and DNA extraction. Culture was attempted on MGIT960 (Becton Dickinson, Cockeysville, USA) automated system in agreement with the manufacturer's instructions.

### Molecular DST

Decontaminated specimens were analyzed for presence of mutations conferring resistance to RIF and INH by the molecular assay GenoType^® ^MTBDR*plus*.

DNA from decontaminated clinical specimens was extracted by thermal lysis and sonication and GenoType^® ^MTBDR*plus *assay was performed in accordance with the manufacture's instructions with minor modifications (as specified below). In brief, 500 μL of decontaminated sample were centrifuged at 13000 rpm at 4°C for 15 minutes; pellet was resuspended in 75 μL of sterile distilled water and mycobacteria were lysed by incubation at 95°C for 30 minutes and sonication for 15 minutes. Five microliters of lysate were used for amplification with the provided biotinylated primers. Two units (instead of 1 as reported in manufacturer's instructions) of hot-start *Taq *DNA polymerase were used in the amplification step. The amplification was performed in a BioRad iCycler (BioRad, Hercules, CA) thermal cycler with a protocol consisting of 1 cycle at 95°C for 15 min (Taq activation cycle), 10 cycles of denaturation at 95°C for 30 sec and primer annealing at 58°C for 2 min, 40 cycles of denaturation at 95°C for 25 sec, primer annealing at 53°C for 40 sec and extension at 70°C for 40 sec, followed by a final extension at 70°C for 8 min.

The GenoType^® ^MTBDR*plus *is based on reverse hybridization between amplicons derived from a multiplex PCR and nitrocellulose-bound wild-type (WT) and mutated (MUT) probes targeting the hotspot region of *rpoB *gene, the codon 315 of *katG *gene and the promoter region of *inhA *gene. The assay also includes amplification controls for each targeted gene, an additional probes for gram-positive GC rich organisms, and a control-probe for MTB complex DNA detection. Hybridisation and washing were performed manually using the TWINCUBATOR^® ^Hybridization Tray (Hain Lifesciences, Nehren, Germany). Strips were interpreted for susceptibility or resistance to RIF and INH according to manufacturer's instructions.

## Results

The GenoType^® ^MTBDR*plus *test was performed on all samples regardless of the sputum smear results. Twenty-one samples out of the 61 enrolled at M0 were not reconfirmed as sputum smear-positive (Figure 1). We obtained a valid test result (based on the hybridisation of the internal controls of the assay) in 60/61 smear-positive and 47/47 smear-negative samples (Table 1).

**Table 1:** GenoType^® ^MTBDR*plus *assay results obtained from sputum smear-positive and smear-negative chronic tuberculosis patients, Burkina Faso

Recruitment^‡ ^(no.)	Smear (no)	GenoType^® ^MTBDR*plus*			Microbiology		
		
		MTB Complex result* (%)	DST result^†^	no. (%)	Culture	DST result^†^	no. (%)
M0 (61)	Positive (40)	Positive (32)	MDR	20 (32.8)	MTB Complex	MDR	18 (29.5)
					MTB Complex	RIF-S, INH-R	2 (3.2)
			RIF-S, INH-R	3 (4.9)	MTB Complex	RIF-S, INH-R	2 (3.2)
					Negative	-	1 (1.6)
			RIF-R, INH-S	1 (1.6)	MTB Complex	MDR	1 (1.6)
			RIF-S, INH-S	8 (13.1)	MTB Complex	RIF-S, INH-S	7 (11.4)
					*M. simiae*	-	1 (1.6)
		Negative (8)	-	8 (13.1)	*M. avium*	-	3 (4.9)
					Other NTM	-	2 (3.2)
					Negative	-	3 (4.9)
	Negative (21)	Positive (11)	RIF-S, INH-R	1 (1.6)	Negative	-	1 (1.6)
			RIF ind, INH-S	1 (1.6)	Negative	-	1 (1.6)
			RIF-S, INH-S	9 (14.8)	MTB Complex	RIF-S, INH-R	1 (1.6)
					*M. intracellulare*	-	1 (1.6)
					Negative	-	7 (11.4)
		Negative (10)	-	10 (16.4)	*M. avium*	-	2 (3.2)
					*M. intracellulare*	-	1 (1.6)
					Negative	-	7 (11.4)
Follow-up (47)	Positive (21)	Positive (14)	MDR	9 (19.1)	MTB Complex	MDR	7 (14.9)
					MTB Complex	RIF-S, INH-R	1 (2.1)
					Negative	-	1 (2.1)
			RIF-R, INH-S	1 (2.1)	MTB Complex	MDR	1 (2.1)
			RIF-S, INH-R	1 (2.1)	Negative	-	1 (2.1)
			RIF-S, INH-S	3 (6.4)	MTB Complex	RIF-S, INH-R	1 (2.1)
					Negative	-	2 (4.3)
		Negative (6)	-	6 (12.8)	*M. avium*	-	1 (2.1)
					*M. intracellulare*	-	1 (2.1)
					Other NTM	-	1 (2.1)
					Negative	-	3 (6.4)
		Ind (1)	-	1 (2.1)	MTB Complex	RIF-S, INH-S	1 (2.1)
	Negative (26)	Positive (6)	MDR	1 (2.1)	*M. avium*	-	1 (2.1)
			RIF-S, INH-R	1 (2.1)	*Nocardia spp.*	-	1 (2.1)
			RIF-S, INH-S	4 (8.5)	Negative	-	4 8.5)
		Negative (20)	-	20(42.6)	*M. avium*	-	1 (2.1)
					Other NTM	-	1 (2.1)
					Negative	-	18 (38.3)

* MTB Complex, *Mycobacterium tuberculosis *Complex.

^† ^DST, Drug susceptibility testing; MDR, Multidrug-resistant; RIF-S, Rifampin susceptible; RIF-R, Rifampin resistant; INH-S, Isoniazid susceptible; INH-R, Isoniazid resistant; ind, indeterminate

^‡ ^M0, month 0; Follow-up, from month 1 to month 30.

### Sputum smear-positive sample analysis

Out of 61 samples reconfirmed as positive and tested (40 at M0 and 21 collected during therapy), 46 cases were found positive for MTB DNA confirming an active or past disease. Twenty nine out of 46 (63.0%) were identified as MDR by GenoType^® ^MTBDR*plus *test. The percentage increased to 67.4% when two cases identified as RIF monoresistant were included. Eleven samples showed a molecular profile suggestive of a TB strains susceptible to both RIF and INH and 4 showed only mutations associated with INH resistance. Fourteen samples resulted MTB DNA negative. In 1 case we were not able to obtain the expected amplification result.

Liquid cultures were attempted from all the sputum smear-positive samples. Forty out of 46 samples positive for MTB DNA grew a TB strain, 1 grew a non-tuberculous mycobacteria (NTM, *M. simiae*) and 5 resulted negative. Analysis of the line probe assay test from those 6 cases showed absence of mutations conferring resistance to RIF in 3 of them (including the NTM case), one MDR case, and two INH monoresistant patients. These last cases was after 3 months after the beginning of the therapy with category IV regimen.

Further testing on the isolated strains by DST performed on MGIT960 confirmed 25 out of 29 MDR cases previously recognized by molecular test and also identified as MDR the 2 RIF monoresistant cases. Three MDR cases at the molecular test were INH monoresistant by DST. Sequencing analysis of these strains allowed characterization of all the mutations within the hotspot region of *rpoB *gene (M515I+H526N, L533P, and H526N respectively).

Fourteen samples showed a GenoType^® ^MTBDR*plus *test negative for MTB complex probe, and in 8 cases the corresponding cultures grew an NTM strain.

### Sputum smear-negative samples analysis

Despite the fact that we did not re-confirm as smear-positive 21 patients enrolled at M0, we included these samples in the molecular analysis. Twenty-six patients enrolled during therapy and resulting smear-negative were also included in the study (Table 1).

We obtained a GenoType^® ^MTBDR*plus *test positive for MTB complex DNA in 17/47 samples (36.2%); 13 of them didn't show any mutations on the test (27.8%), 1 sample resulted MDR and 2 showed mutations associated with resistance to INH. In one case the molecular test resulted indeterminate for the *rpoB *gene.

Positive mycobacterial cultures were obtained from 3/17 (17.6%) samples; one grew MTB complex (monoresistant to INH) and two of them grew NTMs (*M. avium, M. intracellulare*). Two samples identified as INH mono-resistant by the molecular assay resulted to be a culture-negative and a *Nocardia spp. *strain, respectively.

The MTBDR*plus *resulted negative for presence of MTB DNA in the remaining 30 samples (63.8%), from five samples we cultured NTMs (3 *M. avium*, 1 *M. fortuitum*, 1 *M. intracellulare*).

### Frequency of mutations

Frequency of mutations in MTB complex positive samples identified as resistant to RIF and/or INH by the MTBDR*plus *assay are summarized in Table 2. Concerning smear-positive samples, amongst the 31 RIF-R detected cases, the most represented mutation is the D516V substitution in the hotspot region of *rpoB *gene, involved in resistant phenotype in 41.9% (13/31) of cases. Other mutations recognized were H526D (16.1%, 5/31), H526Y (16.1%, 5/31), and S531L (9.7%, 3/31), respectively. In 16.1% (5/31) of cases, resistance to RIF was identified by the lack of hybridization for WT probes in the analyzed region of *rpoB *gene. Two of them were confirmed as RIF-R by DST. Three cases bearing the following mutations in *rpoB *(M515I+H526N, L533P, and H526N respectively) resulted RIF-S by DST and were further investigated. Minimum inhibitory concentration (MIC) evaluation was performed on the strains. The two strains harbouring L533P and H526N, respectively, despite resulting RIF susceptible, showed a slight increase in the MIC value (0.5 μg/mL). The strain carrying the 515I+H526N substitutions showed a MIC of 2 μg/mL.

**Table 2:** MTBDR*plus*-detected mutation frequency for RIF-R and INH-R on 63 MTB complex-positive samples, Burkina Faso

Smear result (no.)	GenoType^® ^MTBDR*plus*				
	
	Drug susceptibility testing result	*rpoB*	*katG*	*inhA*	no. (%)
Positive (46)	MDR	D516V	S315T(1)	t-8c	5 (10.8)
		D516V	S315T(1)	t-8a	2 (4.3)
		D516V	S315T(1)	wt	5 (10.8)
		H526D	S315T(1)	wt	4 (8.7)
		H526D	S315T(2)	wt	1 (2.2)
		H526Y	S315T(1)	t-8c	2 (4.3)
		H526Y	S315T(1)	t-8a	1 (2.2)
		H526Y	S315T(1)	wt	2 (4.3)
		S531L	S315T(1)	c-15t	1 (2.2)
		S531L	S315T(1)	wt	1 (2.2)
		No wt 3–4–7	S315T(1)	c-15t	1 (2.2)
		No wt 7	S315T(1)	wt	3 (6.5)
		No wt 8	S315T(1)	wt	1 (2.2)
	RIF-R, INH-S	D516V	wt	wt	1 (2.2)
		S531L	wt	wt	1 (2.2)
	RIF-S, INH-R	wt	S315T(1)	wt	3 (6.5)
		wt	S315T(1)	t-8a	1 (2.2)
	RIF-S, INH-S	wt	wt	wt	11 (23.9)
Negative (17)	MDR	D516V	S315T(1)	c-15t	1 (5.8)
	RIF-S, INH-R	wt	S315T(1)	t-8a	1 (5.8)
		wt	S315T(1)	wt	1 (5.8)
	RIF indeter, INH-S	indeter.	wt	wt	1 (5.8)
	RIF-S, INH-S	wt	wt	wt	13 (76.5)

* MDR, Multidrug-resistant; RIF-S, Rifampin susceptible; RIF-R, Rifampin resistant; INH-S, Isoniazid susceptible; INH-R, Isoniazid resistant; wt, wild-type; indeter, indeterminate.

Concerning INH resistance, all cases identified by the molecular assay (n 33) were carrying a S315T substitution in *katG *gene. In 39.4% (13/33) of cases, a mutation affecting the promoter region of *inhA *gene was also detected. Observed frequency of mutation in this region was 21.2% (7/33) t-8c, 12.1% (4/33) t-8a, and 6.1% (2/33) c-15t, respectively. In 20 out of 33 cases (60.6%) no mutation was identified in the promoter region of *inhA *gene.

Frequency of mutations observed in sputum smear-negative samples are also reported in Table 2.

## Discussion

Conventional methods for mycobacteriological culture and DST are slow and labour intensive, requiring sequential procedures for isolation of mycobacteria from clinical specimens, identification of MTB complex, and *in vitro *testing of strain susceptibility to anti-TB drugs. During this time patients may be prescribed inadequate treatment, thus fuelling the development and/or spread of drug resistance. Novel technologies for rapid detection of anti-TB drug resistance have therefore become a priority in TB research and development, and molecular line probe assays focused on rapid detection of RIF resistance (alone or in combination with INH) are now commercially available.

In June 2008, concomitantly with the first published meta-analysis on MTBDR line probe assays [24], WHO has made public the policy statement on molecular line probe assays for rapid screening of patients at risk of MDR-TB [25]. However, adoption of line probe assays does not eliminate the need for conventional culture and DST capability, as culture remains necessary for definitive diagnosis of TB in sputum smear-negative patients, while conventional DST of second line drugs is required to diagnose XDR-TB. Moreover, culture and DST are still essential for patient follow-up during treatment.

The aim of our study was to evaluate the molecular assay GenoType^® ^MTBDR*plus *for detecting DR-TB directly in sputum specimens as a means to provide a more accurate management of chronic TB patients in Burkina Faso. Our study demonstrates that the GenoType^® ^MTBDR*plus *assay can be used to identify MDR-TB cases correctly in the absence of culture facilities in TB patients classified as "chronic" according to internationally accepted criteria. Moreover, this study strongly suggests that sputum smear-microscopy as the only criteria to identify chronic TB patients is inadequate because of the risk of false positives.

The use of the GenoType^® ^MTBDR*plus *molecular test, with further confirmation from culture and DST, allowed readjustment of patients' treatment in over half of the cases studied: 1) patients who were classified and treated as MDR-TB cases harbouring RIF- and INH-S strains (n 24); 2) patients negative for MTB complex DNA among those enrolled at M0 or remaining smear-positive during follow-up (n 24); 3) patients with a NTM infection (n 13). In addition, the correct classification of patients allowed to reconsider their need for hospitalization in MDR-TB wards limiting nosocomial exposure to MDR-TB.

The GenoType^® ^MTBDR *plus *assay performed directly on clinical specimens is less expensive than culture-based DST. We recommend that cost-effectiveness in applying the test should be carefully evaluated in each setting taking into account specific situations (e.g. TB and HIV prevalence), technical skills available on site, and frequency of mutations among resistant samples to evaluate the sensitivity of the molecular assay.

More data is needed to validate the use of molecular assays for rapid detection of drug resistance to key anti-TB drugs such as RIF and INH under routine conditions.

Our study found that the GenoType^® ^MTBDR *plus *yielded interpretable results in more than 99% of the cases, among both smear-positive and smear-negative sputum samples.

Reported sensitivities for RIF- and INH- resistance for the GenoType^® ^MTBDR *plus *are ≥ 97% and ≥ 90%, respectively [24]. Also specificities are accounted to be ≥ 99% for both RIF- and INH- resistances. In our study, 4 samples showed INH susceptibility by molecular line probe assay, while these were identified as INH-resistant by culture based DST. A possible explanation is the lower sensitivity of the test on INH resistance since the GenoType^® ^MTBDR *plus *targets only 2 of the several genomic regions involved in determining INH-resistance [26].

Three samples designated as MDR-TB by GenoType^® ^MTBDR *plus *assay, were not confirmed to be RIF resistant on culture-based DST. Further investigation on these 3 cases identified mutations in the *rpoB *gene (M515I+H526N, L533P, and H526N respectively). Discrepancies between culture and molecular DST can be interpreted by studying the MICs of the corresponding strains. In fact, strains carrying the substitution L533P in *rpoB *gene have been demonstrated to have different MIC values (from 0.5–1.0 μg/mL up to 32 μg/mL) [27,28]. Further evaluation on the three strains carrying substitutions in *rpoB *gene allowed to identify increased MIC values.

Our study had several limitations. Discrepant results in sputum smear microscopy in Burkina Faso and Italy could be due to differences in the sampling time and in recruitment of patients by peripheral centers with suboptimal capacity in smear microscopy.

Discrepant GenoType^® ^MTBDR*plus*-positive and culture-negative samples may be justified in two ways. First, some samples were collected and transported to Italy under sub-optimal conditions and this may have affected their culture results [29]. Second, most of the culture-negative samples harboured DNA from MTB susceptible strains by GenoType^® ^MTBDR*plus*. For these cases we speculate that standard TB treatment was effective and prescription of WHO Category IV regimen unnecessary.

One case with negative culture and with a positive MDR profile on molecular assay may be due to the fact that this case was sampled six months after the beginning of the therapy with category IV regimen. In fact, we observed during treatment follow-up that the molecular assay became negative between month 3 and month 6 in patients under effective treatment (data not shown).

Two sputum smear-negative samples harbouring NTM (*M. intracellulare*, *M. avium*) were identified as MTB complex positive by the GenoType^® ^MTBDR*plus*. This may be due to successful treatment that contributed to select microorganisms not belonging to the MTB complex.

Results obtained in our sample suggest that in Burkina Faso the frequency of mutations involved in RIF resistance differs from that of other settings. The commonest mutation is the D516V substitution in the hotspot region of *rpoB *gene, detected entirely in 43.8% (14/32) of RIF resistant cases. The S531L mutation is responsible of resistant phenotype in only 9.4% (3/32) in our study, whereas this substitution affects the majority of RIF resistant strains in most of other countries worldwide [26]. In this study we observed that mutations in the promoter region of *inhA *gene had a minor role in conferring resistance to INH. As these data were obtained from chronic patients undergoing two cycles of standard first-line drugs regimen, we believe that low-level resistance mutations have favoured secondary selections for high-level resistance mutations such as S315T substitution in the *katG *gene [30], and we recommend further studies be conducted.

The molecular test correctly identifies as MTB-complex negative all the patients in which the smear positivity was probably due to the presence of NTMs as suggested by culture results. The addition of a Mycobacteria genus specific probe could address this problem improving the clinical usefulness of the test, especially in high HIV prevalence settings.

As shown in other studies the molecular assay has been successfully performed on contaminated cultures to obtain drug susceptibility data [17]. This provides important drug resistance data in settings where culture and culture DST testing are not conducted correctly as confirmed by quality control systems.

## Conclusion

In conclusion, our results demonstrate the usefulness of the GenoType^® ^MTBDR *plus *assay in identifying MDR-TB cases (among patients satisfying the chronic TB definition), in the absence of culture facilities. The molecular assay improved the management of chronic cases by offering timely access to more appropriate anti-TB regimens, and this could limit the emergence of resistance due to the misuse of antibiotics, and reduce public health expenses. Guidelines and algorithms for line probe assays use should be available to improve quality and cost-effective use of molecular assays in TB control, and be specifically evaluated in local settings. We recommend that an external quality assurance system should exist for all laboratories performing the test.

## Competing interests

The authors declare that they have no competing interests.

## Authors' contributions

PM carried out and analyzed molecular assay and sequencing data and drafted the manuscript. NS participated in study design and in the local coordination during the study. She helped to draft the manuscript. MD has made substantial contributions to the acquisition and analysis of data. He participated in design of the study and helped to draft the manuscript. MO has made substantial contributions to acquisition and analysis of data. GB has made substantial contributions to acquisition and analysis of data. GP carried out microscopy and microbiology analyses in Italy and contributed in drafting the manuscript. GBM has made substantial contributions in conceiving, analysing and writing the manuscript. AM participated in the design of the study and helped to draft the manuscript. DMC participated in the design of the study and conceived, and analysed the manuscript. All authors read and approved the final manuscript.

## Pre-publication history

The pre-publication history for this paper can be accessed here:

http://www.biomedcentral.com/1471-2334/9/142/prepub
